# (2R,6R)-Hydroxynorketamine Treatment of Rats Exposed to Repetitive Low-Level Blast Injury

**DOI:** 10.1089/neur.2022.0088

**Published:** 2023-03-30

**Authors:** Georgina Perez Garcia, Gissel M. Perez, Rita De Gasperi, Miguel A. Gama Sosa, Alena Otero-Pagan, Rania Abutarboush, Usmah Kawoos, Jonathan K. Statz, Jacob Patterson, Carolyn W. Zhu, Patrick R. Hof, David G. Cook, Stephen T. Ahlers, Gregory A. Elder

**Affiliations:** ^1^ Research and Development Service, James J. Peters Department of Veterans Affairs Medical Center, Bronx, New York, USA.; ^2^ Department of Neurology, Icahn School of Medicine at Mount Sinai, New York, New York, USA.; ^3^ Department of Psychiatry, Icahn School of Medicine at Mount Sinai, New York, New York, USA.; ^4^ General Medical Research Service, James J. Peters Department of Veterans Affairs Medical Center, Bronx, New York, USA.; ^5^ Department of Neurotrauma, Naval Medical Research Center, Silver Spring, Maryland, USA.; ^6^ The Henry M. Jackson Foundation for the Advancement of Military Medicine, Inc., Bethesda, Maryland, USA.; ^7^ Nash Family Department of Neuroscience and Friedman Brain Institute, Icahn School of Medicine at Mount Sinai, New York, New York, USA.; ^8^ Department of Geriatrics and Palliative Care, Icahn School of Medicine at Mount Sinai, New York, New York, USA.; ^9^ Mount Sinai Alzheimer's Disease Research Center and Ronald M. Loeb Center for Alzheimer's Disease, Icahn School of Medicine at Mount Sinai, New York, New York, USA.; ^10^ Department of Geriatrics and Palliative Care, Icahn School of Medicine at Mount Sinai, New York, New York, USA.; ^11^ Geriatric Research Education and Clinical Center, VA Puget Sound Health Care System, Seattle, Washington, USA.; ^12^ Department of Medicine, University of Washington, Seattle, Washington, USA.; ^13^ Neurology Service, James J. Peters Department of Veterans Affairs Medical Center, Bronx, New York, USA.

**Keywords:** (2R,6R)-hydroxynorketamine, blast, post-traumatic stress disorder, rat, traumatic brain injury

## Abstract

Many military veterans who experienced blast-related traumatic brain injuries (TBIs) in the conflicts in Iraq and Afghanistan suffer from chronic cognitive and mental health problems, including post-traumatic stress disorder (PTSD). Male rats subjected to repetitive low-level blast exposure develop chronic cognitive and PTSD-related traits that develop in a delayed manner. Ketamine has received attention as a treatment for refractory depression and PTSD. (2R,6R)-hydroxynorketamine [(2R,6R)-HNK] is a ketamine metabolite that exerts rapid antidepressant actions. (2R,6R)-HNK has become of clinical interest because of its favorable side-effect profile, low abuse potential, and oral route of administration. We treated three cohorts of blast-exposed rats with (2R,6R)-HNK, beginning 7–11 months after blast exposure, a time when the behavioral phenotype is established. Each cohort consisted of groups (*n* = 10–13/group) as follows: 1) Sham-exposed treated with saline, 2) blast-exposed treated with saline, and 3) blast-exposed treated with a single dose of 20 mg/kg of (2R,6R)-HNK. (2R,6R)-HNK rescued blast-induced deficits in novel object recognition (NOR) and anxiety-related features in the elevated zero maze (EZM) in all three cohorts. Exaggerated acoustic startle was reversed in cohort 1, but not in cohort 3. (2R,6R)-HNK effects were still present in the EZM 12 days after administration in cohort 1 and 27 days after administration in NOR testing of cohorts 2 and 3. (2R,6R)-HNK may be beneficial for the neurobehavioral syndromes that follow blast exposure in military veterans. Additional studies will be needed to determine whether higher doses or more extended treatment regimens may be more effective.

## Introduction

Public awareness of military-related traumatic brain injury (TBI) increased during the conflicts in Iraq and Afghanistan.^1^ Whereas military-related TBIs occur through various mechanisms, certain means of imparting injury to the brain, in particular blast exposure, is much more common among military service members than in civilian populations. Indeed, in Iraq and Afghanistan, blast exposures caused by exposures to mortars, artillery shells, and improvised explosive devices constituted the major cause of TBI.^1–3^ In addition to in-theatre exposures, there are also concerns over potential adverse effects of subclinical blast exposures.^4–6^ This type of blast exposure, now being referred to as military occupational blast exposure, is common for many service members in combat and non-combat settings.^4^

A history of TBI is frequently found in veterans seeking treatment at Department of Veterans Affairs (VA) mental health clinics.^7^ TBI is closely linked to chronic mental health problems in veterans,^1^ including anxiety, depression, impulsivity, insomnia, and suicidality.^8,9^ A striking feature in the most recent veterans from Iraq and Afghanistan has been the overlap between a history of blast-related mild TBI (mTBI) and clinical symptoms consistent with a diagnosis of post-traumatic stress disorder (PTSD).^1^ The reasons for this association are not yet clear.

Male rats subjected to repetitive low-level blast exposure exhibit chronic cognitive and PTSD-related traits that develop in a delayed manner.^10–15^ These animals model the enduring neurobehavioral syndromes that veterans often suffer after blast-related TBI.^1,16–18^

Ketamine has received considerable attention as a treatment of refractory depression and PTSD.^19,20^ Ketamine is metabolized to several metabolites, including (2R,6R)-hydroxynorketamine [(2R,6R)-HNK].^21^ Like ketamine, (2R,6R)-HNK exerts rapid antidepressant actions and reverses stress-related reactions in a number of pre-clinical models.^22–32^ (2R,6R)-HNK has become of clinical interest because of its better side-effect profile and less abuse potential than ketamine as well as its oral bioavailability.^29,31,33^

Here, we tested whether (2R,6R)-HNK might reverse established PTSD-related behavioral traits in male rats after repetitive low-level blast exposure. We show that (2R,6R)-HNK consistently reversed certain cognitive deficits and anxiety-related traits.

## Methods

### Animals

Adult male Long-Evans rats (250–350 g, 8–9 weeks of age) were obtained from Charles River Laboratories International (Wilmington, MA). Blast or sham exposures were delivered at 10 weeks of age. All animals were received from the vendor at the same time; blast and sham exposures were performed at the same time, and animals were transported together in a single shipment from the Naval Medical Research Center to the Bronx VA. At the Bronx VA, they were housed in the same room under similar housing conditions and underwent behavioral testing together. The study protocol was reviewed and approved by the Institutional Animal Care and Use Committees of the Walter Reed Army Institute of Research (WRAIR)/Naval Medical Research Center and the James J. Peters VA Medical Center. Experiments were conducted in compliance with the Animal Welfare Act and the principles set forth in the Guide for Care and Use of Laboratory Animals (Institute of Laboratory Animals Resources, National Research Council, National Academy Press, 2011).

### Blast overpressure exposure

Rats were exposed to overpressure injury using a shock tube, which simulates the effects of air blast exposure under experimental conditions. The shock tube has a 0.32-m circular diameter and is a 5.94-m-long steel tube divided into a 0.76-m compression chamber separated from a 5.18-m expansion chamber. The compression and expansion chambers are separated by polyethylene terephthalate Mylar® sheets (Du Pont, Wilmington, DE) that control the peak pressure generated. Peak pressure at the end of the expansion chamber was determined with piezoresistive gauges specifically designed for pressure-time (impulse) measurements (Model 102M152; PCB Piezotronics, Depew, NY).

Individual rats were anesthetized using an isoflurane gas anesthesia system consisting of a vaporizer, gas lines, and valves and an activated charcoal-scavenging system adapted for use with rodents. Rats were placed into a polycarbonate induction chamber, which was closed and immediately flushed with 5% isoflurane mixture in air for 2 min. Rats were placed into a cone-shaped plastic restraint device and then placed in the shock tube. Movement was further restricted during the blast exposure using 1.5-cm diameter flattened rubber tourniquet tubing. Three tourniquets were spaced evenly to secure the head region, the upper torso, and lower torso while the animal was in the plastic restraint cone. The end of each tubing was threaded through a toggle and run outside of the exposure cage where it was tied to firmly affix the animal and prevent movement during the blast overpressure (BOP) exposure without restricting breathing.

Rats were randomly assigned to sham or blast conditions and were placed in the shock tube lying prone with the plane representing a line from the tail to the nose of the body in line with the longitudinal axis of the shock tube with the head placed more upstream. Total length of time under anesthesia, including placement in the shock tube and execution of the blast procedure, was typically <3 min. Blast-exposed animals received 74.5-kPa (equivalent to 10.8 psi, duration 4.8 ms, impulse 175.8 kPa*ms) exposures administered one exposure per day for 3 consecutive days. Further details of the physical characteristics of the blast wave are described in Ahlers and colleagues.^34^ Control (sham)-exposed animals were treated identically, including receiving anesthesia and being placed in the blast tube, but did not receive a blast exposure. Within 10 days after the last blast or sham exposure, animals were transported in a climate-controlled van from the WRAIR to the James J. Peters VA Medical Center (Bronx, NY). Animals left in the morning from the WRAIR and arrived in the afternoon of the same day at the James J. Peters VA Medical Center, where all other procedures were performed.

### Animal housing

Animals were housed at a constant 70–72^o^F temperature with rooms on a 12:12-hour light cycle with lights on at 7:00 am. All subjects were individually housed in standard clear plastic cages equipped with Bed-O'Cobs laboratory animal bedding (The Andersons, Maumee, OH) and EnviroDri nesting paper (Sheppard Specialty Papers, Milford, NJ). Access to food and water was *ad libitum*. Subjects were housed on racks in random order to prevent rack position effects. Cages were coded to allow maintenance of blinding to groups during behavioral testing. All cohorts received the same treatment and handling.

### Drug administration

**FIG. 1.**
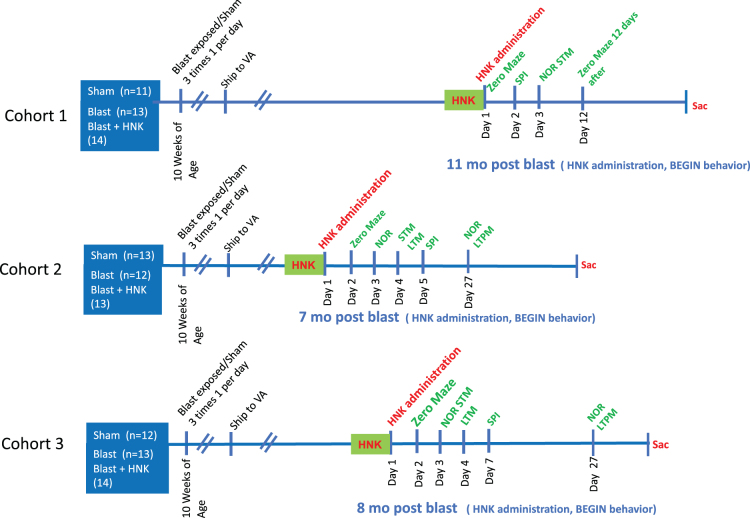
(2R,6R)-HNK treatment of blast-exposed rats. (2R,6R)-HNK, (2R,6R)-hydroxynorketamine; LTM, long-term memory; LTPM, long-term permanent memory; NOR, novel object recognition; SPI, startle pre-pulse inhibition; STM, short-term memory; VA, Department of Veterans Affairs .

(2R,6R)-HNK hydrochloride (Sigma-Aldrich, St. Louis, MO) was dissolved in saline. Blast-exposed subjects were treated with a single dose of 20 mg/kg of (2R,6R)-HNK administered intraperitoneally. Animals were divided into three experimental groups: 1) sham-exposed rats treated with vehicle (sham + veh); 2) blast-exposed rats treated with vehicle (blast + veh); and (3) blast-exposed rats treated with (2R,6R)-HNK (blast + HNK). Three cohorts were studied. Timing of drug administration, in relationship to blast exposure and behavioral testing, is shown in Figure 1. Dose was chosen based on previous studies in rats.^26,35^

### Behavioral testing

Three cohorts of blast-exposed rats and controls were studied. The same protocols were used in each cohort. Number of animals per cohort and the exact timing of testing of each cohort in relationship to blast exposure are described in Figure 1 and Table 1.

**Table 1.** Summary of Results for the Tests Performed and Timing of Each Test in Relation to the Last Blast Exposure

	Cohort 1	Cohort 2	Cohort 3
Time tested	11 months post-blast	7 months post-blast	8 months post-blast
Elevated zero maze (EZM)	**Anxiety phenotype reversed by HNK**	**Partial anxiety phenotype reversed by HNK**	**Anxiety phenotype reversed by HNK**
Acoustic startle pre-pulse Inhibition (SPI)	**Enhanced acoustic startle reversed by HNK**	No phenotype in blast + saline compared to sham + saline	No reversal of enhanced acoustic startle by HNK
Novel object recognition (NOR)	**Impaired object recognition at 1 h reversed by HNK**	**Impaired object recognition at 1 h (STM) and 24 h (LTM) reversed by HNK**	**Impaired object recognition at 1 h (STM) and 24 h (LTM) reversed by HNK**
Delayed effects of HNK	**EZM still partially reversed 12 days after HNK administration**	**Object recognition in NOR still reversed 27 days after HNK administration**	**Object recognition in NOR still reversed 27 days after HNK administration**

Results highlighted in bold indicate tasks in which the blast-induced phenotype was reversed by (2R,6R)-HNK treatment.

HNK, hydroxynorketamine; STM, short-term memory; LTM, long-term memory.

#### Elevated zero maze (EZM)

The apparatus consisted of a circular black Plexiglas runway 121.92 cm in diameter and raised 76 cm off the floor (San Diego Instruments, San Diego, CA). The textured runway itself was 5.08 cm across and divided equally into alternating quadrants of open runway enclosed only by a 1.27-cm lip and closed runway with smooth 15.24-cm walls. All subjects received a 5-min trial beginning in a closed arc of the runway. During each trial, subjects were allowed to move freely around the runway, with all movement tracked automatically by a video camera placed on the ceiling directly above the maze. Data were analyzed by ANYMAZE (San Diego Instruments) yielding measures of total movement time and distance for the entire maze, as well as time spent and distance traveled in each of the individual quadrants. From the quadrant data, measures of total open and closed arc times, latency to enter an open arc, total open arm entries, and latency to completely cross an open arc between two closed arcs were calculated. Subject position was determined by centroid location.

#### Novel object recognition

Rats were habituated to the arena (90 cm length × 60 cm width × 40 cm height) for 20 min, 24 h before training. On the training day, two identical objects were placed on opposite ends of the empty arena, and the rat was allowed to explore the objects freely for 7 min. After a 1-h delay, during which the rat was held in its home cage, one of the two familiar objects (FOs) was replaced with a novel object (NO), and the rat was allowed to freely explore the FO and NO for 5 min to assess short-term memory (STM). After a 24-h delay, during which the rat was held in its home cage, the NO from the STM testing was replaced with a second NO different from the one used during the STM testing. The rat was allowed to freely explore the familiar object (FO) and novel object (NO) for 5 min to assess long-term memory (LTM). Raw exploration times for each object are expressed in seconds. Object exploration was defined as sniffing or touching the object with the vibrissae or when the animal's head was oriented toward the object with the nose placed at a distance of <2 cm from the object.

All sessions were recorded by video camera (Sentech, Carrollton TX) and analyzed with ANYMAZE software (San Diego Instruments). In addition, offline analysis by an investigator blind to the blast-exposed status of the animals was performed. Objects to be discriminated were of different size, shape, and color and were made of plastic or metal material. Objects consisted of a 330-mL soda can, metal box, cup, and plastic tube. All objects were cleaned with 70% ethanol between trials.

#### Pre-pulse inhibition and acoustic startle

Startle magnitude and sensory gating were examined in a 40-trial pre-pulse inhibition assay (San Diego Instruments). Animals were placed in isolation chambers inside closed Plexiglas tubes, each of which was mounted on a platform resting on an accelerometer. After a 5 min habituation period with a 74-dB background white noise, each animal received 40 randomized trials separated by 20–30 sec. Trials consisted of 10 each of background readings taken at 74 dB, startle trials with readings after 40-msec 125-dB tones, pre-pulse inhibition trials where the 125-dB tone was preceded 100 msec earlier by a 20-msec 79-dB tone, and control trials consisting of only the 20-msec 79-dB pre-pulse. On all trials, maximum magnitude of the animal's startle (or other motion) was automatically recorded in 500-msec windows by an accelerometer. Tubes were rinsed clean between animals. Percent pre-pulse inhibition was calculated with the formula 100 – (startle response on acoustic pre-pulse plus pulse stimulus trials/pulse stimulus response alone trials) × 100]. The first startle response was also compared among groups.

### Statistical analysis

Values are expressed as mean ± standard error of the mean. Comparisons were performed using one-way analysis of variance (ANOVA), repeated-measures ANOVA, or two-tailed unpaired *t*-tests. When repeated-measures ANOVA was used, significance was determined using the Greenhouse-Geisser correction.^36^ Statistical tests were performed using the program GraphPad Prism (version 9.4.1; GraphPad Software Inc., Cary, NC) or SPSS statistical software (v27; IBM Corp., Armonk, NY).

## Results

Rats exposed to low-level repetitive BOP injuries using 74.5-kPa exposures delivered once per day for 3 consecutive days (3 × 74.5 kPa) develop a variety of cognitive- and PTSD-related behavioral traits.^10–14^ Multiple cohorts of rats exposed to this protocol have been tested 3–4 months or longer after a single sequence of blast exposures and have consistently exhibited a PTSD-related behavioral phenotype.^10,11,13–15,37^

Figure 1 outlines the experimental design. We treated three cohorts of blast-exposed rats with (2R,6R)-HNK, beginning 7–11 months after blast exposure, a time when the behavioral phenotype is established^10,11,13–15,37^ and a time relevant to military veterans who often present for medical care with already established symptoms. The groups consisted of: 1) sham-exposed rats treated with saline, 2) blast-exposed rats treated with saline, and 3) blast-exposed rats treated with 20 mg/kg of (2R,6R)-HNK. Comparison of sham- + saline-treated controls with blast- + saline-treated animals provided a positive control for development of the blast-related behavioral phenotype. Comparing blast + saline with blast + HNK allowed effects of (2R,6R)-HNK on the blast-related phenotype to be determined. Rats were tested in a subset of behavioral tasks found previously to be informative.^10,11,13–15,37^ Figure 1 shows the tests performed and timing of each test in relation to the last blast exposure and (2R,6R)-HNK. Table.1 summarizes the results.

### Testing of cohort 1

**FIG. 2.**
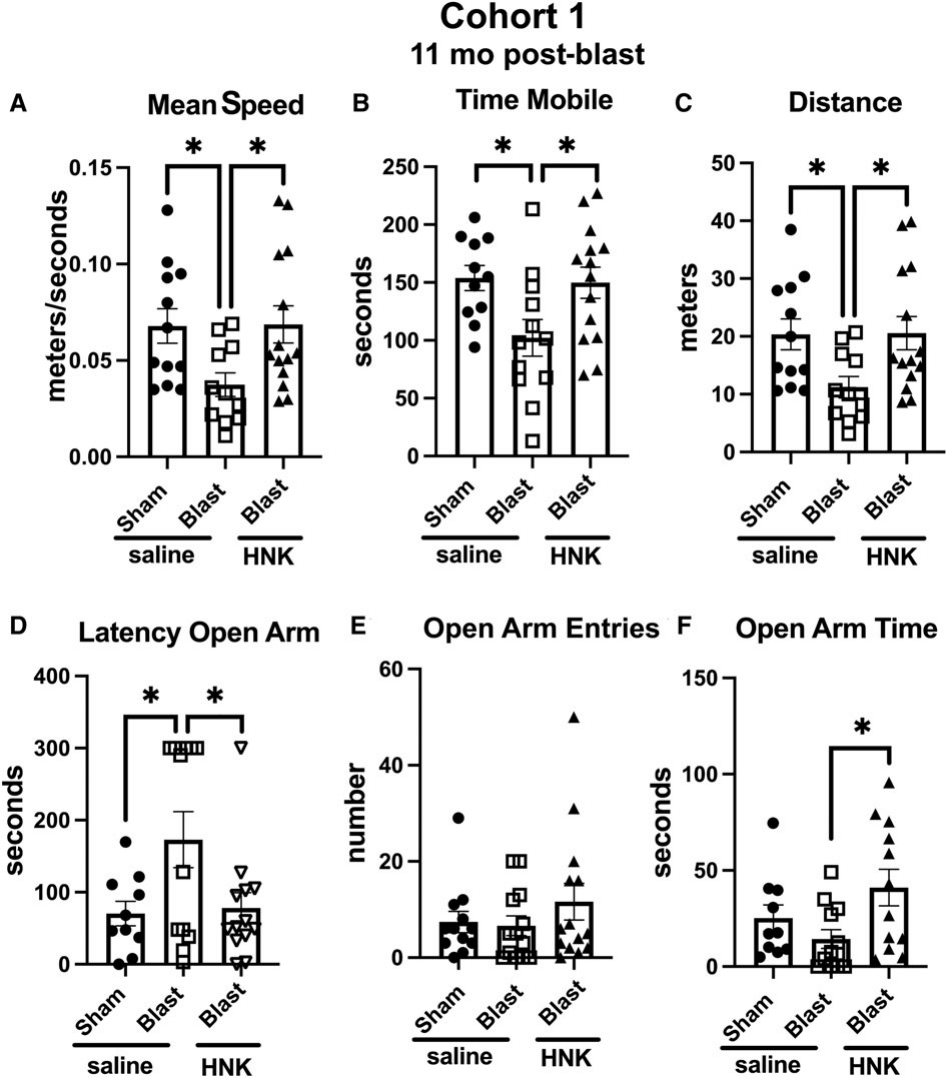
Elevated zero maze (EZM) testing of cohort 1. Blast-exposed rats were treated with saline (*n* = 13) or (2R,6R)-HNK (*n* = 14) and sham-exposed were treated with saline (*n* = 12), as indicated in Figure 1. Rats were tested in the EZM. Shown is mean speed (**A**; one-way ANOVA: *F*_2, 34_ = 3.952, *p* = 0.0286), time spent in motion (**B**; *F*_2, 34_ = 4.269, *p* = 0.0222), distance moved (**C**; *F*_2, 34_ = 3.981, *p* = 0.0280), latency to enter an open arm (**D**; *F*_2,32_ = 4.129, *p* = 0.0254), open arm entries (**E**; *F*_2, 36_ = 0.9055, *p* = 0.4134), and time spent in the open arms (**F**; *F*_2, 34_ = 3.489, *p* = 0.0430). Error bars indicate the standard error of the mean. Asterisks indicate differences between groups after a significant (*p* < 0.05) one-way ANOVA (*p < 0.05, Fisher's LSD). (2R,6R)-HNK, (2R,6R)-hydroxynorketamine; ANOVA, analysis of variance; LSD, least significant difference.

Testing of cohort 1 in an EZM is shown in Figure 2. Compared to sham + saline, blast + saline, moved at a slower speed (*p* < 0.05), spent less time in motion (*p* < 0.05), and moved less distance (*p* < 0.05). As in previous studies, blast + saline exhibited an anxiety phenotype, showing a longer latency to enter an open arm (*p* < 0.05) compared to sham + saline. All of these behaviors in blast-exposed rats treated with saline were reversed by treatment with (2R,6R)-HNK (Fig. 2A–D). Treatment also increased time spent in the open arms by blast + HNK (Fig. 2F) compared to blast + saline (*p* < 0.05).

**FIG. 3.**
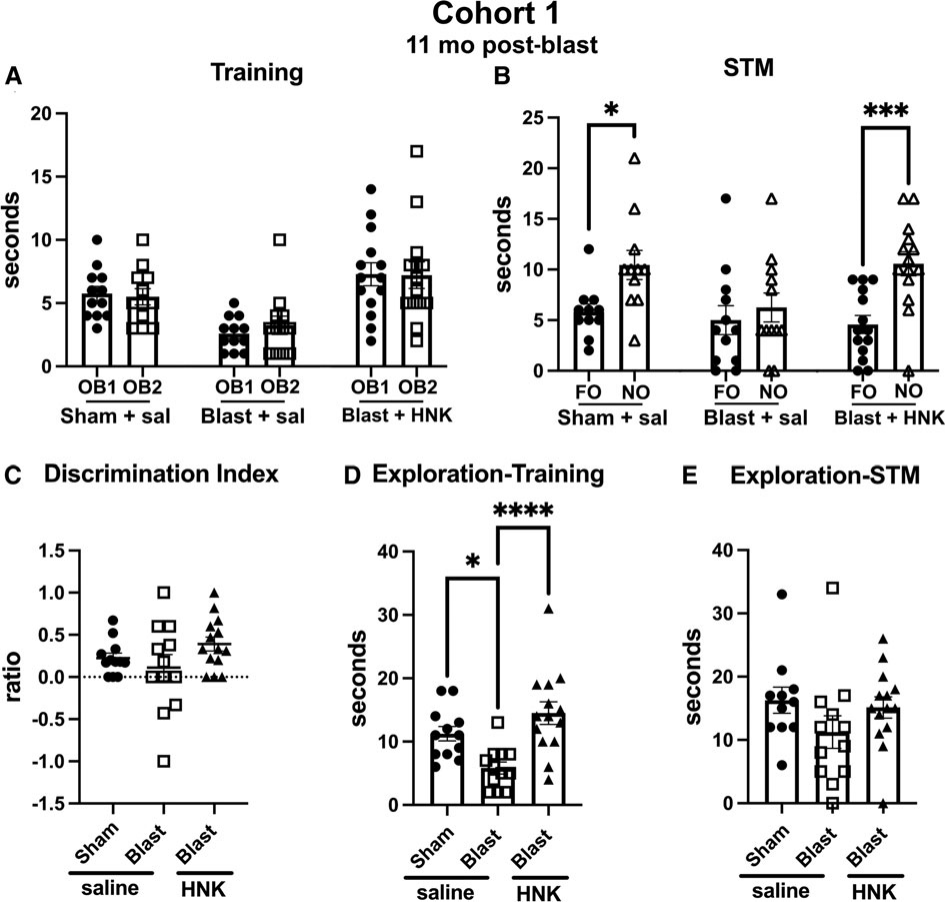
Novel object recognition (NOR) testing of blast-exposed rats from cohort 1 treated with (2R,6R)-HNK. Sham- and blast-exposed rats were treated with saline (*n* = 12 sham, *n* = 13 blast) or blast were treated with (2R,6R)-HNK (*n* = 14). Rats were tested in NOR after treatment as indicated in Figure 1. Panel (**A**) shows time spent exploring the objects (OB1 and OB2) during the training session. Panel (**B**) shows exploration of the previously presented familiar object (FO) compared to the novel object (NO) when presented 1 h (STM) after training. Asterisks indicate values significantly different (*p < 0.05, ***p < 0.001, unpaired *t*-tests). Panel (**C**) shows a discrimination index, which did not significantly differ between the groups. Panels (**D**) and (**E**) show total time spent exploring the objects during the training (one-way ANOVA: *F*_2,35_ = 9.885, *p* = 0.0004) and STM (*F*_2, 34_ = 1.506, *p* = 0.2361) testing (*p < 0.05, ****p < 0.0001, Fisher's LSD). (2R,6R)-HNK, (2R,6R)-hydroxynorketamine; ANOVA, analysis of variance; LSD, least significant difference; STM, short-term memory.

Cohort 1 testing in NOR is shown in Figure 3. During training (Fig. 3A), all groups explored the two objects equally, although blast + saline spent less total time exploring the objects (Fig. 3D) than sham + saline (*p* < 0.05) or blast + HNK (*p* < 0.0001). When presented with the choice between an NO and FO, 1 h later sham + saline explored the NO more than FO (*p* < 0.05) whereas blast + saline explored the NO and FO equally (Fig. 3B). However, blast + HNK explored the FO more than the NO (*p* < 0.001), indicating that the deficits in NOR in the blast-exposed had been rescued by (2R,6R)-HNK.

**FIG. 4.**
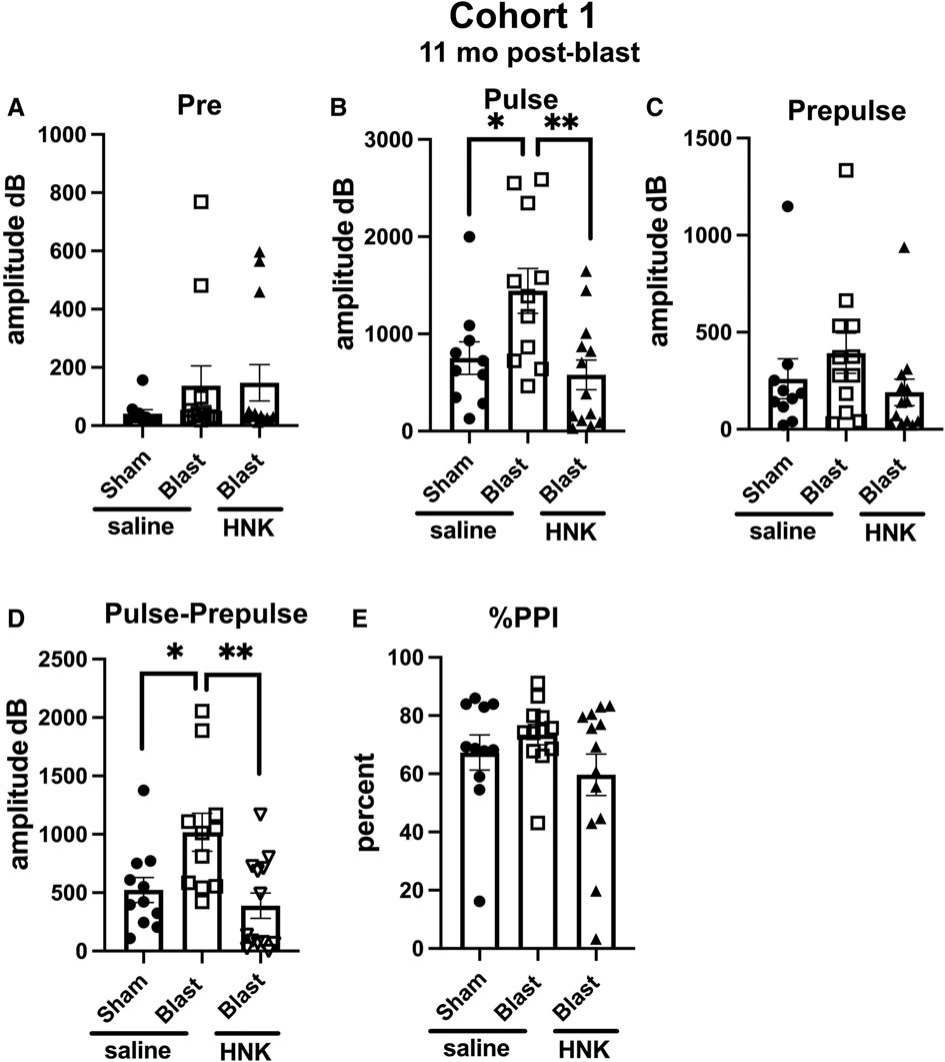
Acoustic startle and sensory gating testing of cohort 1. Shown are background readings (Pre) (**A**; one way ANOVA: *F*_2, 32_ = 0.9456, *p* = 0.3990), acoustic startle response (Pulse) (**B**; *F*_2, 31_ = 6.164, *p* = 0.0056), startle after the pre-pulse (Prepulse) (**C**; *F*_2, 32_ = 1.338, *p* = 0.2767), and the response when the pre-pulse was subtracted from the pulse (Pulse-Prepulse) (**D**; *F*_2, 32_ = 6.779, *p* = 0.0035). Percent pre-pulse inhibition (%PPI) is shown in panel (**E**; *F*_2, 33_ = 1.464, *p* = 0.2459). Asterisks indicate differences between groups after a significant (*p* < 0.05) one-way ANOVA (*p < 0.05, **p < 0.01, Fisher's LSD). (2R,6R)-HNK,(2R,6R)-hydroxynorketamine; ANOVA, analysis of variance; LSD, least significant difference.

Acoustic startle and sensory gating were examined in a pre-pulse inhibition assay (Fig. 4). Resting background did not differ between groups (Fig. 4A). However, blast + saline exhibited an increased acoustic startle (Fig. 4B) compared to sham + saline (*p* < 0.05). The acoustic startle response was, however, normalized in blast + HNK, which did not differ from sham + saline. Groups did not differ in pre-pulse inhibition (Fig. 4C) or percent pre-pulse inhibition (Fig. 4E), although when the pre-pulse was subtracted from the acoustic startle (pulse; Fig. 4D), blast + saline exhibited an increased response compared to sham + saline (*p* < 0.05). This increased response was reversed by (2R,6R)-HNK.

**FIG. 5.**
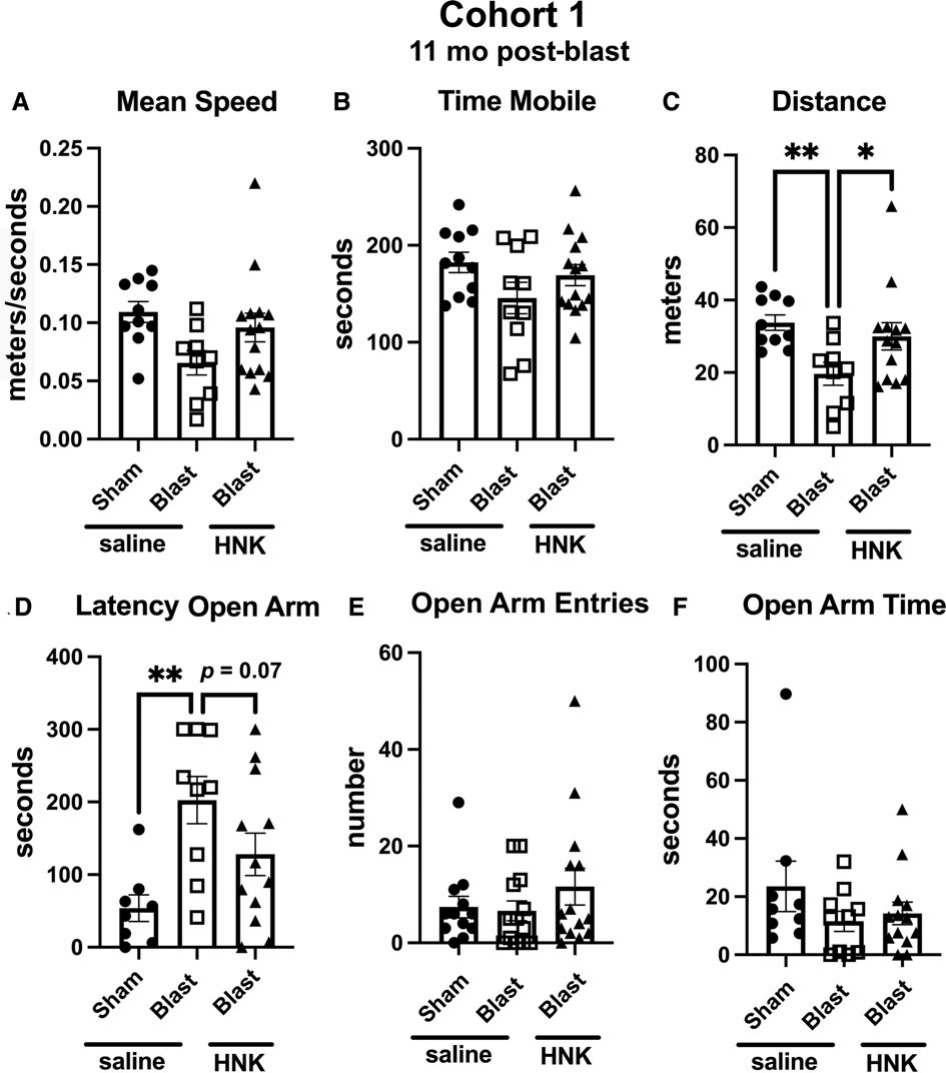
Elevated zero maze (EZM) testing of cohort 1 at 12 days after (2R,6R)-HNK treatment. Rats in cohort 1 were retested in an EZM at 12 days after (2R,6R)-HNK administration. Shown is mean speed (**A**; *F*_2, 30_ = 3.312, *p* = 0.0502), time spent in motion (**B**; *F*_2, 32_ = 2.011, *p* = 0.1504), distance moved (**C**; *F*_2, 29_ = 4.359, *p* = 0.0221), latency to enter an open arm (**D**; *F*_2, 26_ = 5.909, *p* = 0.0077), open arm entries (**E**; *F*_2, 28_ = 2.943, *p* = 0.0692), and time spent in the open arms (**F**; *F*_2, 29_ = 1.223, *p* = 0.3091). Asterisks indicate differences between groups after a significant (*p* < 0.05) one-way ANOVA (*p < 0.05, **p < 0.01). (2R,6R)-HNK, (2R,6R)-hydroxynorketamine; ANOVA, analysis of variance; LSD, least significant difference.

To gain a sense of the length of response to (2R,6R)-HNK, we retested the animals from cohort 1 in an EZM at 12 days after (2R,6R)-HNK treatment. As shown in Figure 5, (2R,6R)-HNK reversed the effects of blast exposure on distance moved (Fig. 5C) and latency to enter an open arm (Fig. 5D), but no longer significantly affected other parameters.

### Testing of cohort 2

**FIG. 6.**
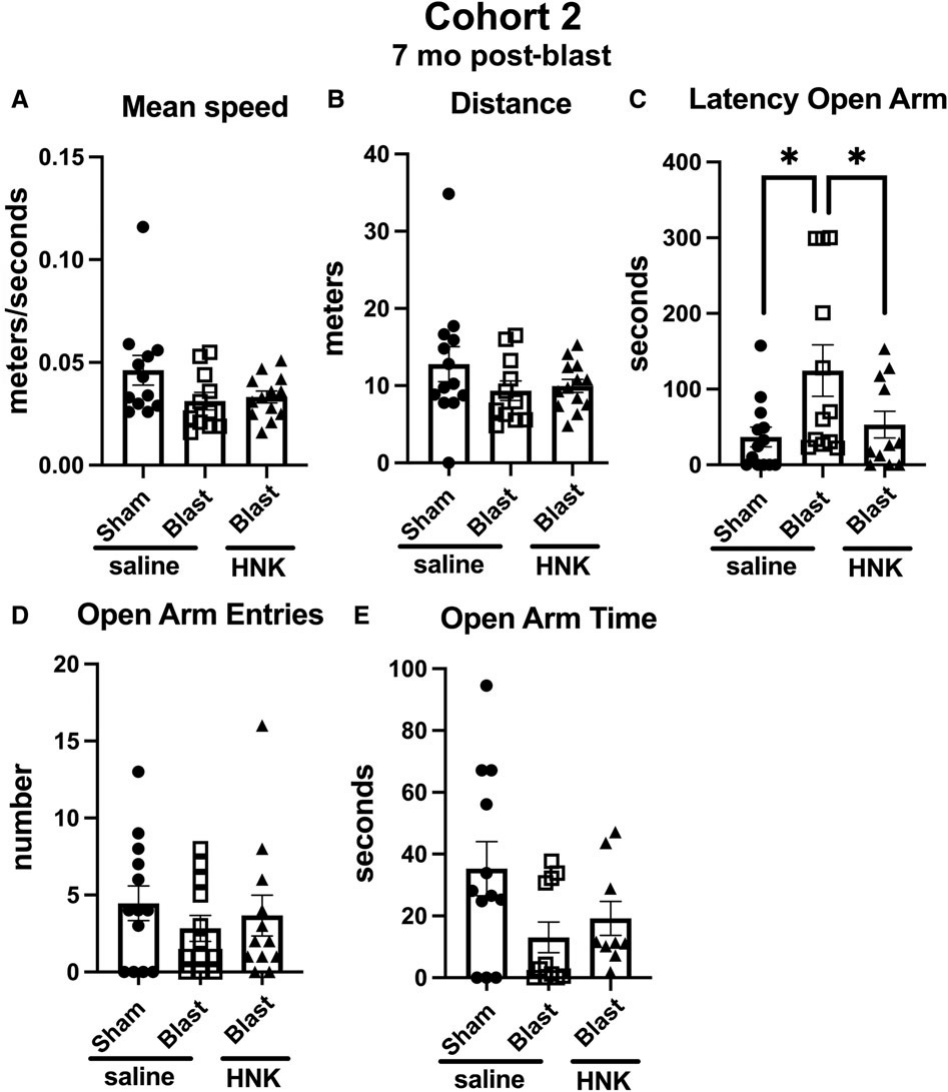
Elevated zero maze (EZM) testing of cohort 2. Blast-exposed rats were treated with saline (*n* = 12) or (2R,6R)-HNK (*n* = 13) and sham-exposed were treated with saline (*n* = 13) as indicated in Figure 1. Shown is mean speed (**A**; one-way ANOVA: *F*_2, 33_ = 2.551, *p* = 0.0933), distance moved (**B**; *F*_2, 34_ = 1.277, *p* = 0.2920), latency to enter an open arm (**C**; *F*_2,33_ = 4.103, *p* = 0.0256), open arm entries (**D**; *F*_2, 34_ = 0.5392, *p* = 0.5881), and time spent in the open arms (**E**; *F*_2, 29_ = 2.943, *p* = 0.0686). Asterisks indicate differences between groups after a significant (*p* < 0.05) one-way ANOVA (*p < 0.05, Fisher's LSD). (2R,6R)-HNK, (2R,6R)-hydroxynorketamine; ANOVA, analysis of variance; LSD, least significant difference.

In cohort 2, the anxiety phenotype in the EZM (Fig. 6) was not as strong in blast-exposed rats treated with saline as in cohort 1. Blast + saline differed from sham + saline only in the latency to enter an open arm (Fig. 6C; *p* < 0.05). However, this effect was rescued in blast + HNK, which exhibited shorter latencies to enter an open arm compared to blast + saline (Fig. 6C; *p* < 0.05), latencies that were not different from sham + saline.

**FIG. 7.**
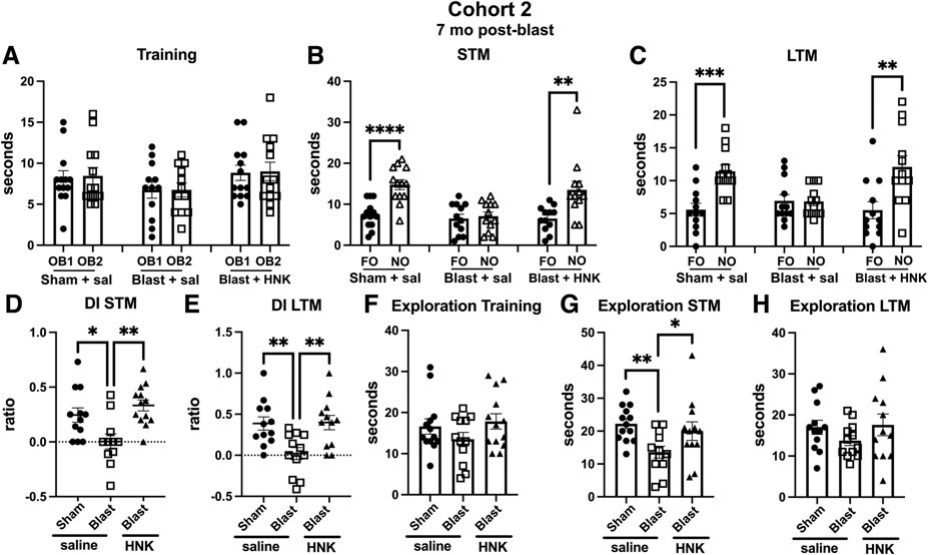
NOR testing of cohort 2. Sham- and blast-exposed rats were treated with saline (*n* = 13 sham, *n* = 12 blast) or blast were treated with (2R,6R)-HNK (*n* = 13). Shown is time spent exploring the objects (OB1 and OB2) during the training session (**A**) or the FO and NO during STM (**B**) and LTM (**C**) testing. Asterisks indicate values significantly different (**p < 0.01, ***p < 0.001, ****p < 0.0001, unpaired *t*-tests). Panels (**D**) and (**E**) show a discrimination index calculated for STM (**D**; one-way ANOVA: *F*_2, 33_ = 7.446, *p* = 0.0021) or LTM (**E**; *F*_2, 33_ = 7.127, *p* = 0.0027) testing. Panels (**F**) to (**H**) show total time spent exploring the objects during training (**F**; *F*_2, 35_ = 1.494, *p* = 0.2384), STM (**G**; *F*_2, 34_ = 4.609, *p* = 0.0169), and LTM (**H**; *F*_2, 33_ = 1.124, *p* = 0.3371) sessions. Asterisks in panels (**D**) to (**H**) (*p < 0.05, **p < 0.01) represent Fisher's LSD performed after a significant ANOVA. (2R,6R)-HNK, (2R,6R)-hydroxynorketamine; ANOVA, analysis of variance; FO, familiar object; LSD, least significant difference; LTM, long-term memory; NO, novel object; NOR, novel object recognition; STM, short-term memory.

When tested in novel object recognition (NOR), results with cohort 2 were similar to cohort 1. All groups explored the objects equally during the training session (Fig. 7A). However, in testing of object recognition memory, which in cohort 2 included testing at 1 h (STM; Fig 7B) and 24 h (LTM; Fig. 7C) after training, whereas sham + saline explored the NOs more than the FOs in both testing sessions, blast + saline explored the NOs and FOs equally. This deficit was reversed in both STM and LTM testing in blast + HNK (Fig. 7B,C). A discrimination index calculated for the STM (Fig. 7D) and LTM (Fig. 7E) sessions confirmed the deficits in blast + saline compared to sham + saline and their rescue in blast-exposed rats treated with (2R,6R)-HNK. Total exploration time did not differ between groups in training (Fig. 7F) or LTM (Fig. 7H) testing sessions. However, in the STM (Fig. 7G), blast + saline explored the objects less than the sham + saline (*p* < 0.01) or blast + HNK (*p* < 0.05).

When acoustic startle and sensory gating were tested in cohort 2, there were no differences between between sham + saline versus blast + saline in acoustic startle response, pre-pulse inhibition, or when the pre-pulse was subtracted from the pulse (data not shown). (2R,6R)-HNK treatment of blast-exposed rats did not affect any of these responses. However, given that blast + saline did not exhibit any phenotype in this assay compared to sham + saline, the (2R,6R)-HNK response is hard to meaningfully interpret.

**FIG. 8.**
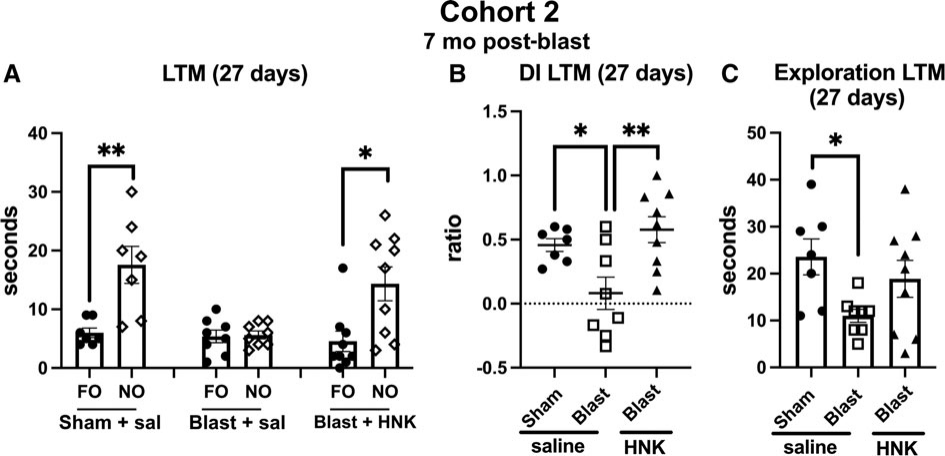
NOR testing of cohort 2 at 27 days after (2R,6R)-HNK administration. A subset of the rats tested in Figure 7 (*n* = 7 sham + veh; 8 blast + veh and 9 blast + HNK) were retested 27 days after (2R,6R)-HNK administration. The NO used in Figure 7 was replaced with an NO different from those used in the previous STM and LTM testing. The FO and NO were retained in similar locations as in Figure 7. Panel (**A**) shows time spent exploring the FO and NO. Asterisks indicate values significantly different (*p < 0.05, **p < 0.01, unpaired *t*-tests). Panel (**B**) shows a discrimination index (one-way ANOVA: *F*_2, 21_ = 6.760, *p* = 0.0056), and panel (**C**) shows total time exploring the objects (*F*_2, 21_ = 3.505, *p* = 0.0486). Asterisks in panels (**B**) and (**C**) (*p < 0.05, **p < 0.01) represent Fisher's LSD performed after a significant ANOVA. (2R,6R)-HNK, (2R,6R)-hydroxynorketamine; ANOVA, analysis of variance; FO, familiar object; LSD, least significant difference; LTM, long-term memory; NO, novel object; NOR, novel object recognition; STM, short-term memory.

To test the length of response to (2R,6R)-HNK, we retested animals from cohort 2 in the NOR task at 27 days after treatment. In this testing, the NO from the LTM session performed earlier (Fig. 8) was replaced with a new NO whereas the FO remained the same. Both objects were placed in the same locations. As shown in Figure 8A, like saline-treated sham animals, blast-exposed animals treated with HNK explored the NO more than the FO (*p* < 0.01, sham + saline; *p* < 0.05, blast + HNK). By contrast, blast + saline explored the NO no more than the FO. When the groups were directly compared using a discrimination index, sham + saline explored the NO compared to FO more than blast + saline (Fig. 8B). This effect was reversed in blast + HNK. Total exploration time (Fig. 8C) was also decreased in blast + saline compared to sham + saline (*p* < 0.05), although HNK treatment did not affect this parameter.

### Testing of cohort 3

**FIG. 9.**
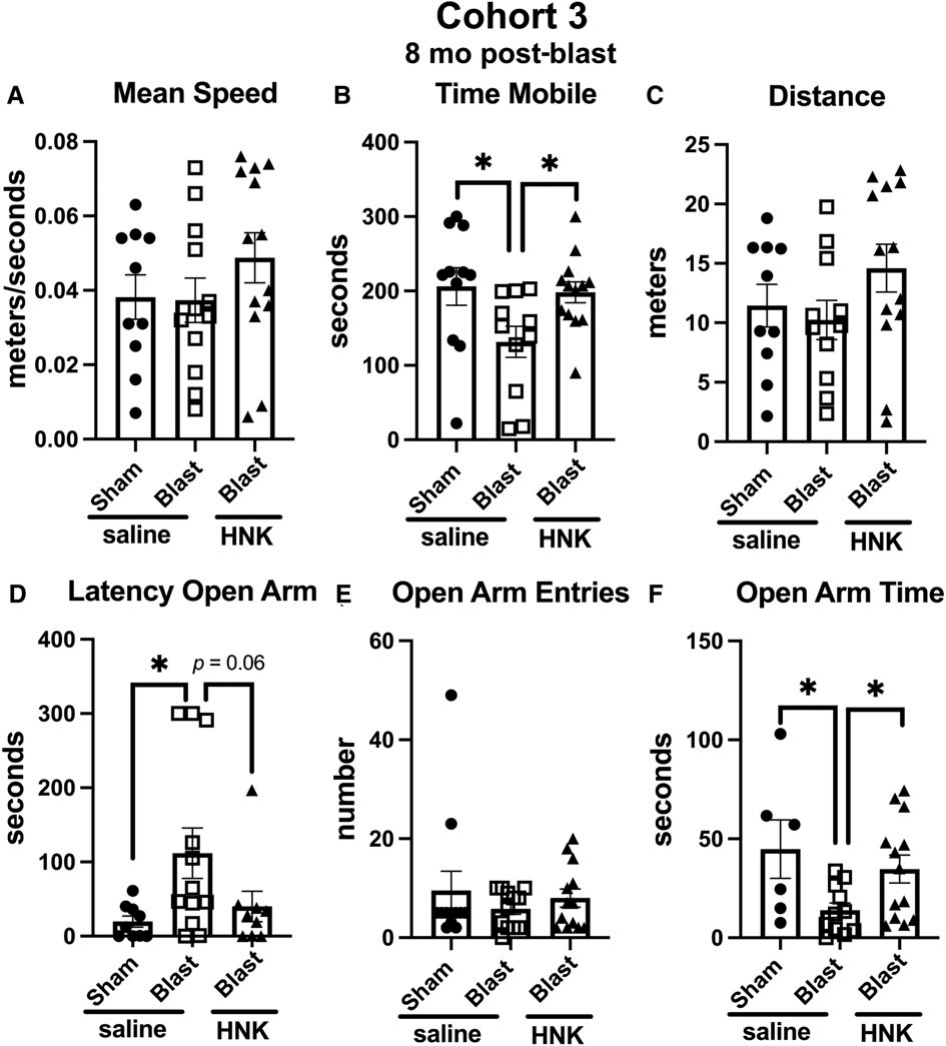
Elevated zero maze (EZM) testing of cohort 3. Blast-exposed rats were treated with saline (*n* = 12) or (2R,6R)-HNK (*n* = 13) and sham-exposed were treated with saline (*n* = 14) as indicated in Figure 1. Shown is mean speed (**A**; one-way ANOVA: *F*_2, 32_ = 1.075, *p* = 0.3532), time in motion (**B**; *F*_2, 32_ = 4.044, *p* = 0.0272), distance moved (**C**; *F*_2, 31_ = 1.564, *p* = 0.2252), latency to enter an open arm (**D**; *F*_2, 27_ = 3.600, *p* = 0.0398), open arm entries (**E**; *F*_2, 34_ = 0.5129, *p* = 0.6033), and time spent in the open arms (**F**; *F*_2, 27_ = 3.802, *p* = 0.0351). Asterisks indicate differences between groups after a significant (*p* < 0.05) one-way ANOVA (*p < 0.05, Fisher's LSD). (2R,6R)-HNK, (2R,6R)-hydroxynorketamine; ANOVA, analysis of variance; LSD, least significant difference.

In cohort 3, as in cohort 2, the anxiety phenotype in the EZM (Fig. 9) was not as strong as in cohort 1. However, blast + saline differed from sham + saline in time in motion (Fig. 9B), latency to enter an open arm (Fig. 9D) and open arm time (Fig. 9E). Treatment with (2R,6R)-HNK reversed blast-induced effects on time in motion (Fig. 9B), open arm time (Fig. 9E) and nearly reverted effects on latency to enter an open arm (p = 0.06; Fig. 9D).

**FIG. 10.**
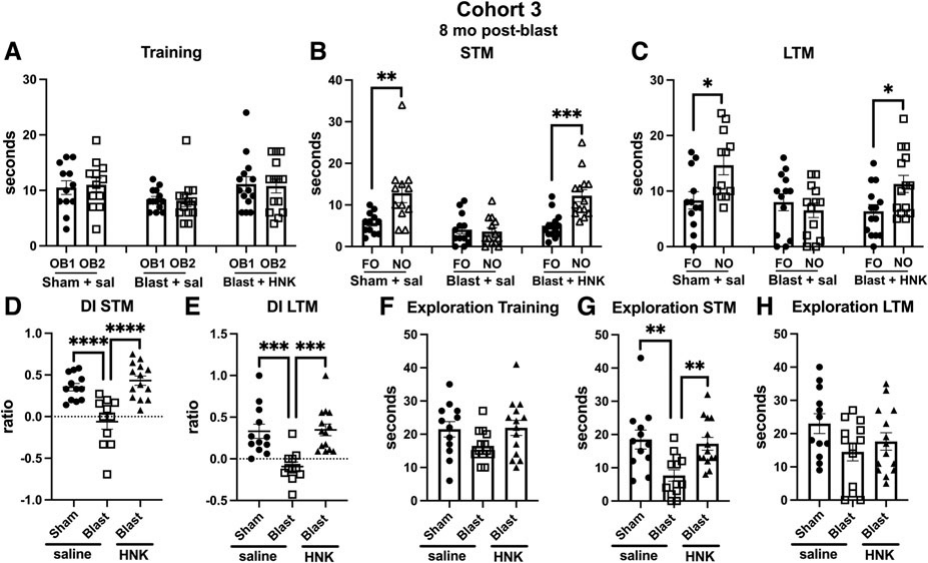
NOR testing of cohort 3. Sham- and blast-exposed rats were treated with saline (*n* = 12 sham, *n* = 13 blast) or blast were treated with (2R,6R)-HNK (*n* = 14). Shown is time spent exploring the objects (OB1 and OB2) during the training session (**A**) or the FO and NO during STM (**B**) and LTM (**C**) testing. Asterisks indicate values significantly different (*p < 0.05, **p < 0.01, ***p < 0.001, unpaired *t*-tests). Panels (**D**) and (**E**) show a discrimination index calculated for STM **(D**; one-way ANOVA: *F*_2, 34_ = 16.40, *p* < 0.0001) and LTM (**E**; *F*_2, 34_ = 11.27, *p* = 0.0002) testing. Panels (**F**) to (**H**) show total time spent exploring the objects during training (**F**; *F*_2, 36_ = 2.189, *p* = 0.1267), STM (**G**; *F*_2, 36_ = 7.363, *p* = 0.0021), and LTM (**H**; *F*_2, 36_ = 2.286, *p* = 0.1162) sessions. Asterisks in panels (**D**) to (**H**) (**p < 0.01, ***p < 0.01, ****p < 0.0001) represent Fisher's LSD performed after a significant ANOVA. (2R,6R)-HNK, (2R,6R)-hydroxynorketamine; ANOVA, analysis of variance; FO, familiar object; LSD, least significant difference; LTM, long-term memory; NO, novel object; NOR, novel object recognition; STM, short-term memory.

When tested in NOR, results with cohort 3 were very similar to cohorts 1 and 2. All groups explored the objects equally during the training session (Fig. 10A). However, in testing of object recognition memory in both STM (Fig. 10B) and LTM (Fig. 10C) testing, sham + saline explored the NOs more than the FOs in both testing sessions whereas blast + saline explored the NOs and FOs equally. This deficit was reversed in both STM and LTM testing in blast-exposed rats treated with (2R,6R)-HNK (Fig. 10B,C). A discrimination index calculated for the STM (Fig. 10D) and LTM (Fig. 10E) sessions confirmed the deficits in blast + saline compared to sham + saline and their rescue in blast-exposed rats treated with (2R,6R)-HNK. Total exploration time did not differ between groups in training (Fig. 10F) or LTM (Fig. 10H) sessions. However, in STM testing (Fig. 10G), blast + saline explored the objects less than the sham + saline (*p* < 0.01), an effect that was rescued by (2R,6R)-HNK treatment.

**FIG. 11.**
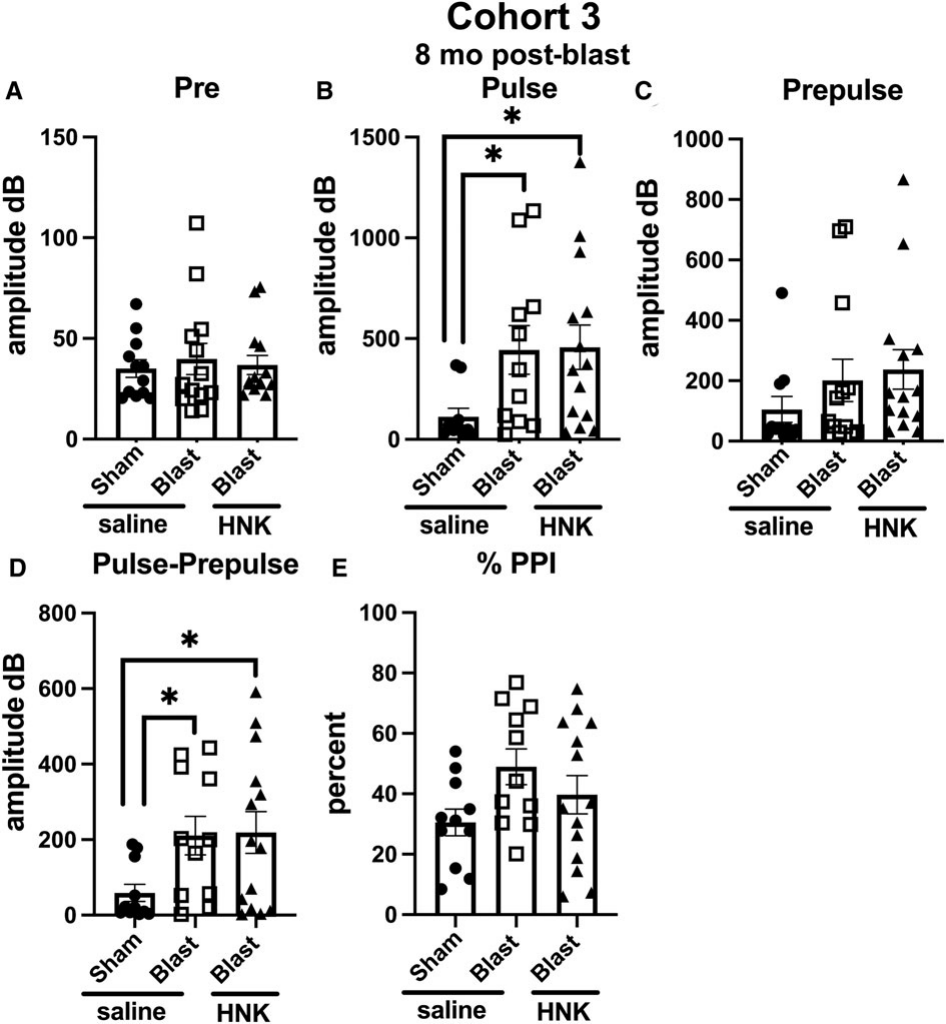
Acoustic startle and sensory gating testing of cohort 3. Sham- and blast-exposed rats were treated with saline (*n* = 12 sham, *n* = 13 blast), or blast were treated with (2R,6R)-HNK (*n* = 14). Shown are background readings (Pre) (**A**; one-way ANOVA: *F*_2, 36_ = 0.1612, *p* = 0.8517), acoustic startle response (Pulse) (**B**; *F*_2, 32_ = 3.313, *p* = 0.0492), startle after the pre-pulse (Prepulse) (**C**; *F*_2, 35_ = 1.121, *p* = 0.3373), and response when the pre-pulse was subtracted from the pulse (Pulse-Prepulse) (**D**; *F*_2, 33_ = 3.446, *p* = 0.0437). Percent pre-pulse inhibition (%PPI) is shown in panel (**E**) (*F*_2, 33_ = 2.323, *p* = 0.1138). Asterisks indicate differences between groups after a significant (*p* < 0.05) one-way ANOVA (*p < 0.05, Fisher's LSD). (2R,6R)-HNK, (2R,6R)-hydroxynorketamine; ANOVA, analysis of variance; LSD, least significant difference.

When acoustic startle and sensory gating were examined in cohort 3 (Fig. 11), resting background did not differ between groups (Fig. 11A). However, blast + saline exhibited an increased acoustic startle (Fig. 11B) compared to sham + saline (*p* < 0.05), but unlike cohort 1, the acoustic startle response was not normalized in blast + HNK, which did not differ from blast + saline (Fig. 11B). Blast + saline also differed from sham + saline when the pre-pulse was subtracted from the acoustic startle (Fig. 11D), an effect that was also not reversed by (2R,6R)-HNK.

**FIG. 12.**
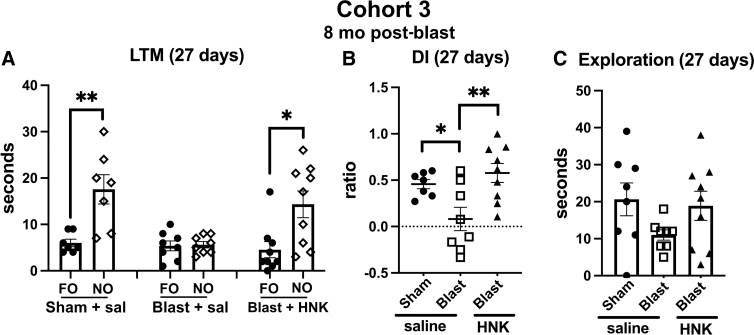
NOR testing of cohort 3 at 27 days after (2R,6R)-HNK administration. A subset of the rats tested in Figure 11 (*n* = 7 sham + veh; 8 blast + veh and 9 blast + HNK) were retested 27 days after (2R,6R)-HNK administration. The NO used in Figure 11 was replaced with an NO different from those used in the previous STM and LTM testing. The FO and NO were retained in similar locations. Panel (**A**) shows time spent exploring the FO and NO. Asterisks indicate values significantly different (*p < 0.05, **p < 0.01, unpaired *t*-tests). Panel (**B**) shows a discrimination index (one-way ANOVA: *F*_2, 21_ = 6.706, *p* = 0.0056), and panel (**C**) shows total spent time exploring the objects (*F*_2, 21_ = 2.013, *p* = 0.1575). Asterisks in panel (**B**) (*p < 0.05, **p < 0.01) represent Fisher's LSD performed after a significant ANOVA. (2R,6R)-HNK, (2R,6R)-hydroxynorketamine; ANOVA, analysis of variance; FO, familiar object; LSD, least significant difference; LTM, long-term memory; NO, novel object; NOR, novel object recognition; STM, short-term memory.

To test the length of response to (2R,6R)-HNK, we retested animals from cohort 3 in the NOR task at 27 days after (2R,6R)-HNK treatment as described for cohort 2 in Figure 10. As shown in Figure 12A, blast + saline explored the NO no more than the FO (Fig. 12A). By contrast, sham + saline animals and blast + HNK explored the NO more than the FO (*p* < 0.01, sham + saline; *p* < 0.05, blast + HNK). When groups were directly compared using a discrimination index, sham + saline explored the NO compared to FO more than the blast + saline (*p* < 0.05; Fig. 12B). This effect was reversed in blast + HNK. Total exploration time did not significantly differ between groups (Fig. 12C).

## Discussion

This study utilized a well-accepted animal model that accurately mimics open field low-level blast exposure and recapitulates translationally relevant mTBI behavioral symptoms associated with human mTBI or subclinical blast exposure in humans.^10,11,13–15,38–44^ Male rats were subjected to exposures of 74.5 kPa (equivalent to 10.8 psi), which represent a level of blast that is transmitted to the brain^45^ but does not produce major gross neuropathological effects.^34^ Blast exposures were delivered at 10 weeks of age, with rats lying prone in the shock tube with the plane representing a line from the tail to the nose of the body directly in line with the longitudinal axis of the shock tube and the head placed more upstream. Head motion was further restricted during the blast exposure to minimize damage from rotational/acceleration injury.^46,47^ The lack of evidence for gross coup/contrecoup injuries supports the relatively mild nature of the brain injury.^10,34^ To mimic the multiple blast exposures commonly experienced by veterans in Iraq and Afghanistan,^48^ rats were subjected to three 74.5-kPa exposures delivered once a day for 3 consecutive days.

Male rats subjected to repetitive low-level blast exposure show cognitive and PTSD-related behavioral traits, including anxiety, enhanced acoustic startle, and impaired recognition memory.^14,15^ These traits develop in a delayed manner, being absent in the first 8 weeks after blast exposure but consistently present 3–4 months and longer after exposure. Once the traits appear, they remain present for more than 1 year after the last blast exposure^10,11,13,14,49^ and are likely present for the lifetime of the animal. These animals thus model the enduring neurobehavioral syndromes that veterans often suffer after blast exposure.^16–18^

Although the traits observed in this study are related to PTSD, the model is not considered a true representation of PTSD because the blast exposures were carried out under general anesthesia, without any additional psychological stressor. Rather, the model proposes that blast exposure alone, even without a psychological stressor, can induce PTSD-related traits, which we have termed “blast-induced PTSD.”^14^ However, it is worth noting that rats in this model exhibit additional anxiety-related changes that persist if exposed to a single predator scent 8 months after the last blast.^12^ This suggests that blast exposure not only induces PTSD-related traits, but also sensitizes the brain to react abnormally to future psychological stressors. These findings have significant implications for how mental health disorders after blast exposure are conceptualized, especially in military veterans with a history of blast-related TBI who have been diagnosed with PTSD. They highlight the importance of distinguishing between physical and psychological trauma in such cases.^50^

We treated three cohorts of blast-exposed rats at 7–11 months after exposure with (2R,6R)-HNK, a time when the blast-related behavioral phenotype is established. This study thus represents a treatment, rather than a prevention study conducted at times relevant to veterans who typically present for medical care with already-established symptoms. Studies with cohort 1 began before the time course of the behavioral phenotype was fully known,^15^ and 11 months was chosen to ensure that treatment would begin within the time frame in which the phenotype had been clearly established.^10–13^ Once the time course of the phenotype's appearance was fully established,^15^ we felt comfortable in moving to earlier time points of 7 and 8 months for cohorts 2 and 3. (2R,6R)-HNK improved behavior in all three cohorts (Table 1). (2R,6R)-HNK rescued deficits in NOR in each cohort. Indeed, recognition memory seemed exquisitely sensitive to rescue by (2R,6R)-HNK.

(2R,6R)-HNK has been mostly studied in models of depression.^22–32,35,51–56^ Although one study reported that (2R,6R)-HNK improved spatial memory deficits in the Wistar-Kyoto rat model of depression,^57^ our study appears to be the first to show that explicit memory can be improved by one administration of (2R,6R)-HNK with beneficial effects maintained for 27 days in the two cohorts in which delayed testing was performed. Deficits in EZM, an anxiety-related task, were also improved in all three cohorts. Acoustic startle was rescued in cohort 1, but not in cohort 3, and results were inconclusive in cohort 2 since the enhanced acoustic startle response was not present in blast-exposed rats treated with vehicle.

Cohorts were studied at different time points after blast exposure, which may account for some of the variability in response to (2R,6R)-HNK between cohorts. However, all three time points were within the range when the behavioral phenotype was present. Why the acoustic startle phenotype was absent in cohort 2 is unclear. However, it does not appear to be attributable to the slightly earlier time at which cohort 2 was studied (7 months post-blast) given that enhanced acoustic startle responses have been observed as early as 3–4 months after blast exposure.^11^

Intuitively, one might expect that at later time points, the phenotype might become more “hardened” and hence difficult to treat. However, cohort 1, which was treated at the latest time point, responded best to treatment. Alternatively, to the degree (2R,6R)-HNK more directly targets central nervous system (CNS) signaling cascades that drive blast-induced behavioral traits, one could argue that the more disturbed these cascades are the more responsive they might be to treatment. Importantly, despite the chronic nature of blast-induced behavioral disturbances, these data strongly suggest that certain causal CNS processes remain sensitive to therapeutic modification when the right pathway is targeted at the right time—even when intervention takes place long after the initiating insults.

Perhaps a more significant factor to be considered than time post-blast is dosing. (2R,6R)-HNK has been widely studied in mice and rats and, similar to ketamine, exerts rapid effects that can persist for weeks.^22–32,35,51–56^ (2R,6R)-HNK has been mostly studied using intraperitoneal dosing. In mice and rats, efficacious doses have been reported over ranges from as little as 0.025 to 30 mg/kg administered intraperitoneally. Most studies have used a range of 10–30 mg/kg. We chose a dose of 20 mg/kg within the mid-range showing effectiveness in rats.^26,27,35,52,55^

The initial choice of 4–6 h to begin the testing of cohort 1 was arbitrary, but based on knowledge that in experimental animals and humans, (2R,6R)-HNK typically exerts rapid effects. We extended this time to 18 h for cohorts 2 and 3 after observing that drug-treated animals in cohort 1 appeared slightly ataxic when EZM testing was conducted on the same day as drug administration. Effects on coordination were not observed in cohorts 2 and 3. Future studies using more extended dosing regimens will be needed to determine whether longer dosing may rescue additional behavioral domains. More studies will also be needed to assess (2R,6R)-HNK's duration of action. It would also be of interest to compare (2R,6R)-HNK directly with ketamine and assess the effects of (2R,6R)-HNK in other behavioral tasks.

Mechanistically, it can only be speculated as to why (2R,6R)-HNK might rescue blast-induced behavioral effects. Ketamine's actions are generally attributed to inhibition of N-methyl-D-aspartate (NMDA) receptors although non-NMDA-related mechanisms also exist.^21,30,58^ (2R,6R)-HNK exerts rapid, sustained behavioral and cellular actions, but is probably spared many of ketamine's adverse side effects because of its low potency to inhibit NMDA reecptor (NMDAR) function relative to ketamine. (2R,6R)-HNK acutely potentiates AMPAR-mediated synaptic transmission in the hippocampus through unknown mechanisms.^59^ Riggs and colleagues^60^ showed that (2R,6R)-HNK selectively enhances excitatory synaptic transmission in the hippocampus through a concentration-dependent increase in glutamate release probability. Notably, these results found that (2R,6R)-HNK leads to a pre-synaptic potentiation that does not require NMDAR activation, is not blocked by NMDAR inhibition, and persists in the presence of tetrodotoxin. HNK-related metabolites also act at other receptors, including α7 nicotinic acetylcholine receptors, which are not affected by ketamine.^21^

In previous work, we studied the effect of BCI-838, a group II metabotropic glutamate receptor (mGluR2/3) antagonist prodrug.^61^ mGluR2/3 antagonists have proneurogenic, procognitive, antidepressant, and anxiolytic activities in many animal models.^61–66^ BCI-838 reversed PTSD-related behavioral traits in blast-exposed rats, improving anxiety- and fear-related behaviors as well as long-term recognition memory concomitant with increased neurogenesis in the dentate gyrus.^11^

Interestingly, studies in mice with knockouts of the metabotropic mGluR2 and mGluR3 receptors have suggested that (2R,6R)-HNK exerts its antidepressant actions through an mGluR2-dependent mechanism.^29^ Collectively, these studies can be seen as suggesting that mGluR2 may be a common site for the action of both BCI-838 and (2R,6R)-HNK. mGluR2 activity has complex effects on a variety of plasticity-relevant signaling cascades, including protein kinase B, extracellular signal–related kinases/mitogen-activated protein kinases, and the mammalian target of rapamycin complex 1 pathway activity, which in turn regulate responses to brain-derived neurotrophic factor as well as glutamate signaling.^67^ Future studies will be needed to examine whether mGluR2-related signal transduction is disturbed after blast exposure and may be the principle site of action of drugs such as BCI-838 and (2R,6R)-HNK.

One limitation of the current study is the lack of inclusion of female rats. Sex differences in outcomes after TBI are well known.^68^ Studies also suggest that female veterans are more likely to report persisting neurobehavioral symptoms and use more outpatient services than their male counterparts.^69^ Sex differences in response to blast exposure have been little studied, although two recent reports have suggested that blast responses in female rats may differ.^70,71^ With the increasing number of female veterans, these studies assume a high importance.

## Conclusion

Based on this pre-clinical study, (2R,6R)-HNK seems promising for the treatment of blast-related cognitive and neurobehavioral syndromes in veterans. Future studies will be needed to optimize dosing regimens and determine length of effect and mechanism of action. Studies comparing (2R,6R)-HNK with ketamine also seem warranted.

## Abbreviations Used

(2R,6R)-HNK: (2R,6R)-hydroxynorketamine
AMPAR: α-amino-3-hydroxy-5-methyl-4-isoxazolepropionic acid receptor
BOP: blast overpressure
CNS: central nervous system
EZM: elevated zero maze
FO: familiar object
LTM: long-term memory
mGluR2/3: group II metabotropic glutamate receptor 2/3
mTBI: mild TBI
NMDA: N-methyl-D-aspartate
NMDAR: NMDA reecptor
NO: novel object
NOR: novel object recognition
PTSD: post-traumatic stress disorder
STM: short-term memory
TBI: traumatic brain injury
VA: Department of Veterans Affairs
WRAIR: Walter Reed Army Institute of Research
